# Mammary tuberculosis mimicking breast cancer: a case report

**DOI:** 10.1186/1752-1947-2-34

**Published:** 2008-02-01

**Authors:** Ioannis Maroulis, Charalambos Spyropoulos, Vasiliki Zolota, Evaggelos Tzorakoleftherakis

**Affiliations:** 1 Department of Surgery, University Hospital of Patras, Rion, Greece; 2 Department of Pathology, University Hospital of Patras, Rion, Greece

## Abstract

**Introduction:**

The incidence of tuberculosis is rising worldwide and rare manifestations of the past are seen more often nowadays. Mammary tuberculosis is a rare clinical entity, often mimicking breast cancer or abscesses of benign or malignant origin. Clinical awareness is necessary during diagnostic work-up for establishing the correct diagnosis and treatment.

**Case presentation:**

We present a case of breast tuberculosis diagnosed in a 73 year old woman at our institution. The patient presented with a palpable mass of the right breast with clinical, laboratory and mammographic findings indicative of breast carcinoma. The patient underwent lumpectomy and sentinel lymph node biopsy. Frozen section of the tumor and the sentinel node revealed "granulomatous inflammation", while gross examination confirmed the diagnosis of tuberculous mastitis. The patient received anti-tuberculosis therapy for six months with no side effects or any further complications.

**Conclusion:**

Breast tuberculosis is an obscure disease often mistaken for carcinoma or pyogenic abscess of the breast, especially if well-defined clinical features are absent. A high index of suspicion is required because the disease can usually be treated conservatively with current antituberculous modalities while surgical intervention is reserved for rare cases only.

## Introduction

The incidence of tuberculosis is sharply rising in developing and developed countries and rare extrapulmonary manifestations of the past can pose challenges in clinical practice. This may be due in part to the increasing number of geriatric patients, especially those with immunosuppression, as well as due to the development of drug resistant strains of Mycobacterium tuberculosis [1,2].

The clinical signs of mammary tuberculosis can be insidious and nonspecific and often simulate signs of breast carcinoma. Mammary tuberculosis usually affects young, multiparous, lactating women although it may also be seen in males in 4.5% of cases [3]. The breast can be the primary site but more commonly, tuberculosis spreads to the breast through the lymphatic system from axillary, mediastinal or cervical lymph nodes, or directly from underlying structures such as the ribs. Most commonly the disease presents as a lump in the central or the upper outer quadrant of the breast while multiple lumps are less frequent. The borders of the lump are usually irregular while fixation of the lesion to the skin, the underlying muscle or even to the chest wall often poses clinical problems in differentiation from breast carcinoma.

The aim of this report is to detail our experience of the difficulties in diagnosing breast tuberculosis, especially in the absence of other specific clinical signs, and to emphasize the impact of anti-tuberculosis chemotherapy and the minor role of surgery.

## Case presentation

A 73-year-old woman, with an unremarkable medical history, was admitted to hospital complaining of a palpable lump located in her right breast. Upon physical examination, a firm mass in the upper outer quadrant of the right breast was found with co-existing edema and erythema of the skin. No evident axillary lymphadenopathy was present. All vital signs, as well as blood and urine analysis and chest X-ray, were normal. Tumor marker analysis revealed that CA-125 was mildly elevated (65 U/ml), while CA 19-9, CA 15-3, a-FP and CEA levels were all within normal limits.

**Figure 1:**
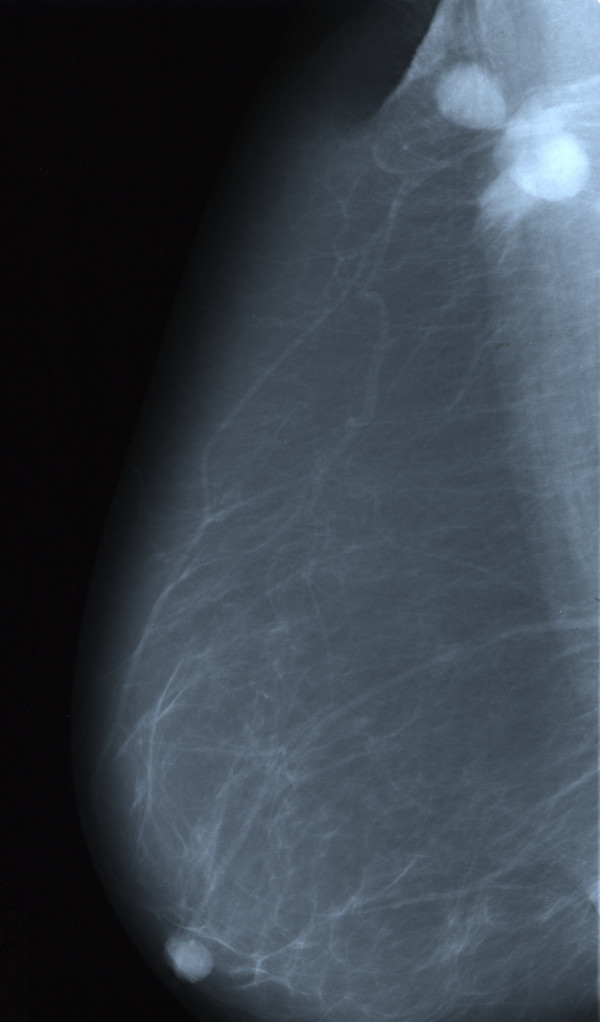
Mammogram showing an abnormal mass in the upper outer quadrant of the right breast. Two axillary lymph nodes are identified.

Mammography was performed and indicated the presence of a mass 1.2 cm in diameter, with abnormal borders located close to the axillary process of the right breast, accompanied by two regional lymph nodes of 1.5 and 2 cm respectively (Figure 1). According to the American College of Radiology Breast Imaging and Reporting Data Systems (BI-RADS), the probability of malignancy was high (category 5). Ultrasonographic findings were similar to that seen on mammography while thoracic CT examination did not reveal any pathology in the lung parenchyma or mediastinum.

Based on the physical examination and laboratory data, a preliminary diagnosis of breast cancer was made and the patient underwent lumpectomy and sentinel lymph node biopsy.

**Figure 2:**
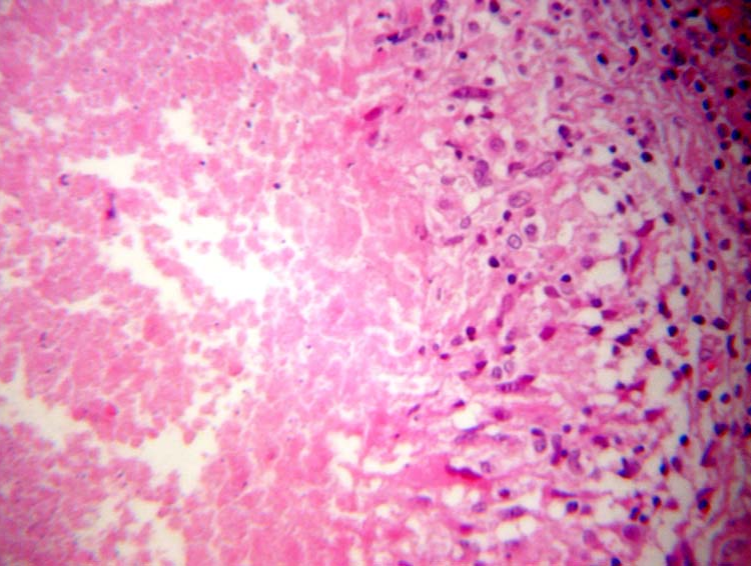
Granuloma composed of a central hypocellular necrotic area and peripherally arranged epithelioid histiocytes.

**Figure 3:**
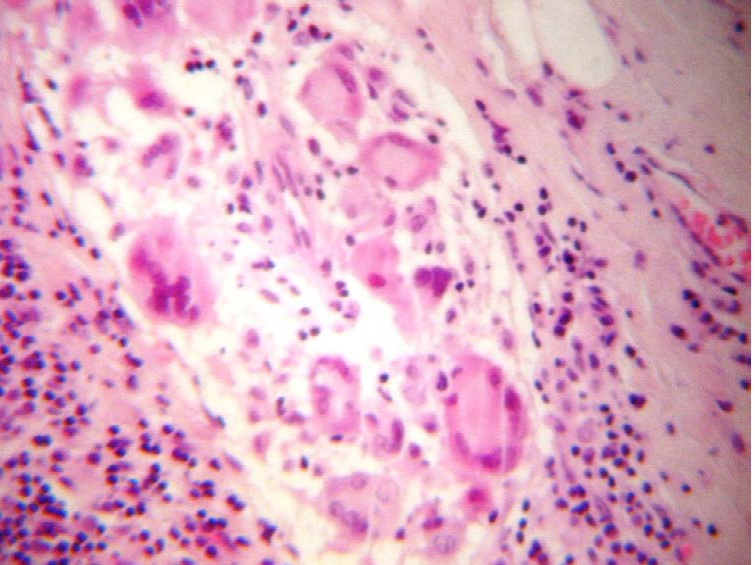
Granuloma with central acellular necrosis and the presence of many giant cells.

Frozen section of the specimen revealed "granulomatous inflammation", while gross examination of the tumor, which measured 1.8 cm in greatest diameter, revealed the presence of a multinodular, fleshy mass which microscopically consisted of fibrous and lymphoid tissue infiltrated by large epithelioid granulomas (Figure 2) with central acellular necrosis and many giant cells (Figure 3). The same morphologic appearance was identified in the sentinel lymph node which measured 0.9 cm in greatest diameter. Special (acid fast) stains failed to demonstrate microorganisms within the necrosis.

The patient had an uncomplicated postoperative course and after the final histopathology report, which established the diagnosis of mammary tuberculosis, underwent a complete work-up including computed tomography of the abdomen, to rule out other foci of tuberculosis, and received anti-tuberculosis therapy with daily doses of 300 mg isoniazid., 600 mg rifampicin, 1500 mg pyrazinamide and 10 mg pyridoxine for six months. No side effects or any complications have been recorded to date.

## Discussion

Breast tuberculosis is a rare clinical entity with incidence ranging from 0.1% in developed countries, to 0.3 – 5% in endemic regions [4]. The disease is more frequently seen in women between 20 and 50 years of age, especially among multiparous and lactating females where the breast is more sensitive to infection and trauma. Tuberculous mastitis may be primary, although this is extremely rare [5], or secondary as a result of hematogenous spreading, retrograde spread from axillary lymph nodes or direct extension from the lung, pleura, mediastinum and articular lesions [5,6]. Clinical presentation is extremely variable, often presenting as round nodular lumps mainly in the upper outer quadrant of the breast [7]. The mass is usually covered with indurated tissue, often with fistula formation, but is rarely associated with pain and breast discharge. In its advanced form, breast tuberculosis is characterized by invasion of the skin, with skin and nipple retraction creating the peau d'orange sign.

Based on radiological and clinical characteristics the disease can be divided into three forms: nodular, diffuse and sclerosing. The nodular form is characterized by a circumscribed lesion in the breast with an oval tumor shadow on mammography, a finding which can rarely be differentiated from breast cancer. The diffuse form of the disease, also known as disseminated tuberculosis mastitis, is characterized by multiple tuberculous foci of the breast which often cause multiple ulcerations and discharging sinuses on the skin; this form simulates inflammatory breast cancer on mammographic findings. The sclerosing form of the disease is more frequently seen in elderly women and is characterized by an excessive fibrotic process. The older McKeown and Wilkinson [8] classification of breast tuberculosis also included tuberculous mastitis obliterans and acute military tubercular mastitis, two forms of the disease which are only of historical importance today.

In the case of our patient, clinical examination failed to differentiate breast carcinoma from tuberculous mastitis. The advanced age of the patient, the non-specific findings of mammography and ultrasonography, as well as a low index of suspicion resulted in an incorrect preliminary diagnosis. Additionally, no fine needle aspiration biopsy or core needle biopsy was performed prior to surgery, which could have raised the possible diagnosis of breast tuberculosis. The patient underwent surgical intervention. Anti-tuberculosis therapy, consisting of the same regimen used in pulmonary tuberculosis [9,10], was applied only after the final histopathological report was received.

## Conclusion

Although breast tuberculosis is considered a rare entity, clinical awareness is essential when treating non-specific breast abnormalities, particularly in regions of the world where tuberculosis is endemic. Diagnosis can be established by fine needle aspiration cytology or histology while antitubercular therapy represents the mainstay of treatment, avoiding unnecessary surgical intervention. Surgery is reserved for selected refractory cases only [11,12].

## Competing interests

The author(s) declare that they have no competing interests.

## Authors' contributions

I.M. conceived of the study, C.S. participated in its design, data collection and helped to draft the manuscript, V.Z. performed the histopathology study and E.T. participated in the coordination of the study. All authors read and approved the final manuscript.

## Consent

Written informed consent was obtained from the patient for publication of this case report and any accompanying images. A copy of the written consent is available for review by the Editor-in-Chief of this journal.
